# Drug transporter and oxidative stress gene expression in human macrophages infected with benznidazole-sensitive and naturally benznidazole-resistant *Trypanosoma cruzi* parasites treated with benznidazole

**DOI:** 10.1186/s13071-019-3485-9

**Published:** 2019-05-24

**Authors:** Jair Téllez, Ibeth Romero, Alvaro José Romanha, Mario Steindel

**Affiliations:** 1 Laboratorio de Protozoologia, Departamento de Microbiologia, Imunologia e Parasitologia, Centro de Ciências Biológicas, Universidade Federal de Santa Catarina, Florianópolis, SC Brazil; 2 Vicerrectoría de Investigaciones, Universidad Manuela Beltrán, Bogotá, Cundinamarca Colombia; 3 Programa de Ciencias Básicas, Universidad Manuela Beltrán, Bogotá, Cundinamarca Colombia

**Keywords:** Chagas disease, Host–pathogen interactions, *Trypanosoma cruzi*, Uptake, Benznidazole

## Abstract

**Background:**

Chagas disease is a potentially life-threatening disease caused by the protozoan parasite *Trypanosoma cruzi.* Current therapeutic management is limited to treatment with nitroheterocyclic drugs, such as nifurtimox (NFX) and benznidazole (BZ). Thus, the identification of affordable and readily available drugs to treat resistant parasites is urgently required worldwide. To analyse the effects of BZ on human macrophage gene expression, a quantitative PCR (qPCR) array analysis was performed using drug transporter and oxidative stress pathway genes to compare the gene expression profiles of human differentiated THP-1 macrophage (THP-1 MΦ) cells infected or not with benznidazole-sensitive (CL Brener) and naturally benznidazole-resistant (Colombiana) *T. cruzi* parasites followed by treatment with BZ.

**Results:**

The gene expression analysis indicated that the expression levels of 62 genes were either up- or downregulated at least 3-fold in the host upon infection with CL Brener and BZ treatment, of which 46 were upregulated and 16 were downregulated. Moreover, the expression level of 32 genes was altered in THP-1 MФ cells infected with Colombiana and treated with BZ, of which 29 were upregulated and 3 were downregulated. Our results revealed that depending on the specific condition, human THP-1 MΦ cells infected with *T. cruzi* strains with sensitive or resistant phenotypes and treated with BZ expressed high mRNA levels of *AQP1*, *AQP9* and *ABCB1* (*MDR1*) compared to those of the control cells.

**Conclusions:**

Our findings suggest that the proteins encoded by *AQP1*, *AQP9* and *ABCB1* may be implicated in benznidazole detoxification. Therefore, studies on gene expression are required to better understand the host response to pathogens and drug treatment integrated with functional and metabolic data to identify potentially novel targets for the treatment of this important and neglected tropical disease.

**Electronic supplementary material:**

The online version of this article (10.1186/s13071-019-3485-9) contains supplementary material, which is available to authorized users.

## Background

Chagas disease is a neglected tropical disease that affects millions of people worldwide and represents a major public health problem. Acute infection control strategies depend on chemotherapy. Since the early 1960s, BZ (N-benzyl-2-nitroimidazole acetamide) has been the drug used for Chagas disease treatment, especially in Central and South American countries [1–3]. Therapeutic schemes based on BZ have been widely accepted in the acute phase of infection by *Trypanosoma cruzi*; however, marked side effects associated with low specificity and systemic toxicity have been reported [4, 5].

Nitroheterocyclic compounds, such as nifurtimox (NFX) and BZ, generally act as prodrugs that need to be activated to assert their cytotoxic effects against *T. cruzi* parasites [6, 7]. BZ activation has been demonstrated to involve type I nitroreductase (NTR) enzymes present in parasites but absent in humans, as well as in the reduction and therefore activation of BZ [8, 9]. Type I NTRs catalyse the reduction of nitroheterocyclic compounds within the parasite and produce a series of metabolites to yield 4,5-dihydroxyimidazole to release glyoxal, promoting damage to macromolecules such as DNA and forming adducts with proteins, DNA and small molecules, as in the case of glutathione [8]. However, in mammalian cells, BZ is metabolized by the reduction of a nitro group to an amino group by a type II NTR [10]. As a result of the re-oxidation process, enzymatic processing of BZ leads to the formation of reactive intermediates, such as a nitro anion radical (R-NO_2_^−^) and the formation of reactive oxygen species (ROS) and reactive nitrogen species (RNS) [6]. This process increases the toxicity of BZ towards both the parasite and the host cells [11, 12].

Drug treatment of intracellular infections requires chemotherapeutic agents to enter and interact inside the host cell to exert their effects, reaching an ideal concentration to eliminate the causal agent. Once inside the cell, drugs may be exposed to different biotransformation processes, which determine their efficacy and toxicity [13]. Thus, BZ has been reported to induce changes in the gene activation of drug transporters in host cells, especially *P-gp* and *MRP2*. ABC transporters affect the therapeutic efficacy of BZ by increasing the efflux and/or decreasing the intracellular accumulation of the drug [14].

To provide an initial assessment of potential drug transporters and oxidative stress-related pathways involved in the toxic effects of BZ in host cells, we studied the expression of specific drug transporters and oxidative stress genes in human macrophages infected with *T. cruzi* strains with different BZ sensitivity phenotypes followed by treatment with BZ.

## Methods

### Parasite cultures

Epimastigotes of the *T. cruzi* CL Brener clone (sensitive phenotype) and the *T. cruzi* Colombiana (resistant phenotype) strain, previously characterized by others authors as benznidazole-sensitive or naturally benznidazole-resistant parasites [15–17] were used in the present study. Here, resistance is defined as the ability of the parasite to differentially survive the effects of BZ exposure *in vitro* in comparison with the sensitive control. Parasites were maintained in culture using liver infusion tryptose medium (LIT) as previously described [18]. *T. cruzi* culture-derived trypomastigotes were obtained by *in vitro* infection of THP-1 macrophage-like cells (ATCC) using previously described conditions [18]. Briefly, differentiated THP-1 macrophages were infected with *T. cruzi* trypomastigotes at a parasite to cell ratio of 3:1, and infection was allowed to proceed for 2 h in FBS-RPMI medium at 37 °C and 5% CO_2_. Free parasites were removed by washing 2–3 times with serum-free RPMI medium. After 72 h at 37 °C under 5% CO_2_, trypomastigotes were collected from the culture supernatant, centrifuged at 600×*g* for 30 min, and then left under the same conditions for 3 h. The number of viable trypomastigotes was determined by counting in a Neubauer chamber, and viable trypomastigotes were used for further assays.

### Human THP-1-derived macrophages

The human monocytic cell line THP-1 (ATCC TIB202), derived from an acute monocytic leukaemia, was cultured and differentiated into macrophages as previously described [19]. THP-1-derived macrophages (THP-1 Mϕ) were used to evaluate the gene expression profile in THP-1 Mϕs infected with *T. cruzi* benznidazole-sensitive and naturally benznidazole-resistant strains and then treated with BZ.

### *Trypanosoma cruzi* infection and benznidazole treatment

THP-1 Mϕ cells were infected with *T. cruzi* trypomastigotes of the CL Brener clone and Colombiana strain, followed by a 14 h incubation (5% CO_2_, 37 °C) to allow differentiation into intracellular amastigotes. Infected cells were treated for 48 h with BZ at concentrations ranging from 3 to 100 μM to determine the IC_50_ for both strains by sigmoidal regression analysis (variable slope response) using GraphPad Prism 5 software (GraphPad Inc., San Diego, CA, USA). To evaluate the gene expression mRNA levels in the treated groups, 13 μM BZ (the IC_80_ for the sensitive phenotype being equivalent to the IC_60_ for the resistant phenotype) was used to treat THP-1 Mϕ cells. Controls (infection-free and drug-free macrophages) were included in all assays. Samples were collected and stored in TRIzol (Invitrogen, Sao Paulo, Brazil) for RNA extraction, PCR array and RT-qPCR assays. In parallel, identically infected and drug-treated cells seeded in chamber slides were evaluated to assess their level of infection. The number of amastigotes per cell (parasite load) was determined by randomly counting 300 cells per well through conventional microscopy under 100× magnification in Giemsa-stained preparations; the percentage of parasite survival and parasitic index were determined as previously described by Sereno et al. [20].

### RNA extraction and first-strand cDNA synthesis

Total RNA was isolated with the RNeasy Mini Kit (Qiagen, Maryland, USA) following the manufacturer’s instructions. Total RNA was quantified using a PicoDrop P200 spectrophotometer (PicoDrop Technologies, Hinxton, UK). First strand cDNA was synthesized with an RT^2^ First Strand kit (SABiosciences, Maryland, USA) in reaction volumes of 20 μl, using 70 ng of total RNA as previously described [21]. The cDNA was diluted to 111 μl by adding RNase-free water and stored at − 20 °C until further use.

### RT^2^ Profiler™ PCR arrays of THP-1 Mϕ cells and PCR array data analysis

cDNA was mixed with RT2 SYBR Green/ROX qPCR Mastermix (SABiosciences) to assess the gene expression level of 168 genes using the RT2 Profiler PCR Arrays kit, which includes 84 human drug transporter genes (catalogue number PAHS-070Z, SABiosciences) and 84 human oxidative stress genes (catalogue number PAHS-065Z, SABiosciences), following the manufacturer’s instructions. The arrays contain a panel of standard controls to monitor genomic DNA contamination as well as first-strand synthesis and real-time PCR efficiency. Data were analysed by the ΔΔCt method as described previously [21].

### RT-qPCR validation

The arrays were validated by RT-qPCR using mRNA from the group described above. Three representative genes of the array (MT3, AQP1 and AQP7) were chosen based on their putative significance in drug detoxification and antioxidant defence involvement, and RT-qPCR was performed with the same sets of primers used in the gene array assays (SABiosciences).

### Statistical analyses

Differences were examined by two-way analysis of variance (ANOVA) followed by Bonferroni *post hoc* tests, as described in the figure legends. Analyses were performed with GraphPad Prism 5, and *P*-values of < 0.05 were considered significant.

## Results and discussion

### Effect of benznidazole on the intracellular viability of benznidazole-sensitive and naturally benznidazole-resistant *T. cruzi* strains in human THP-1 MΦ cells

**Fig. 1:**
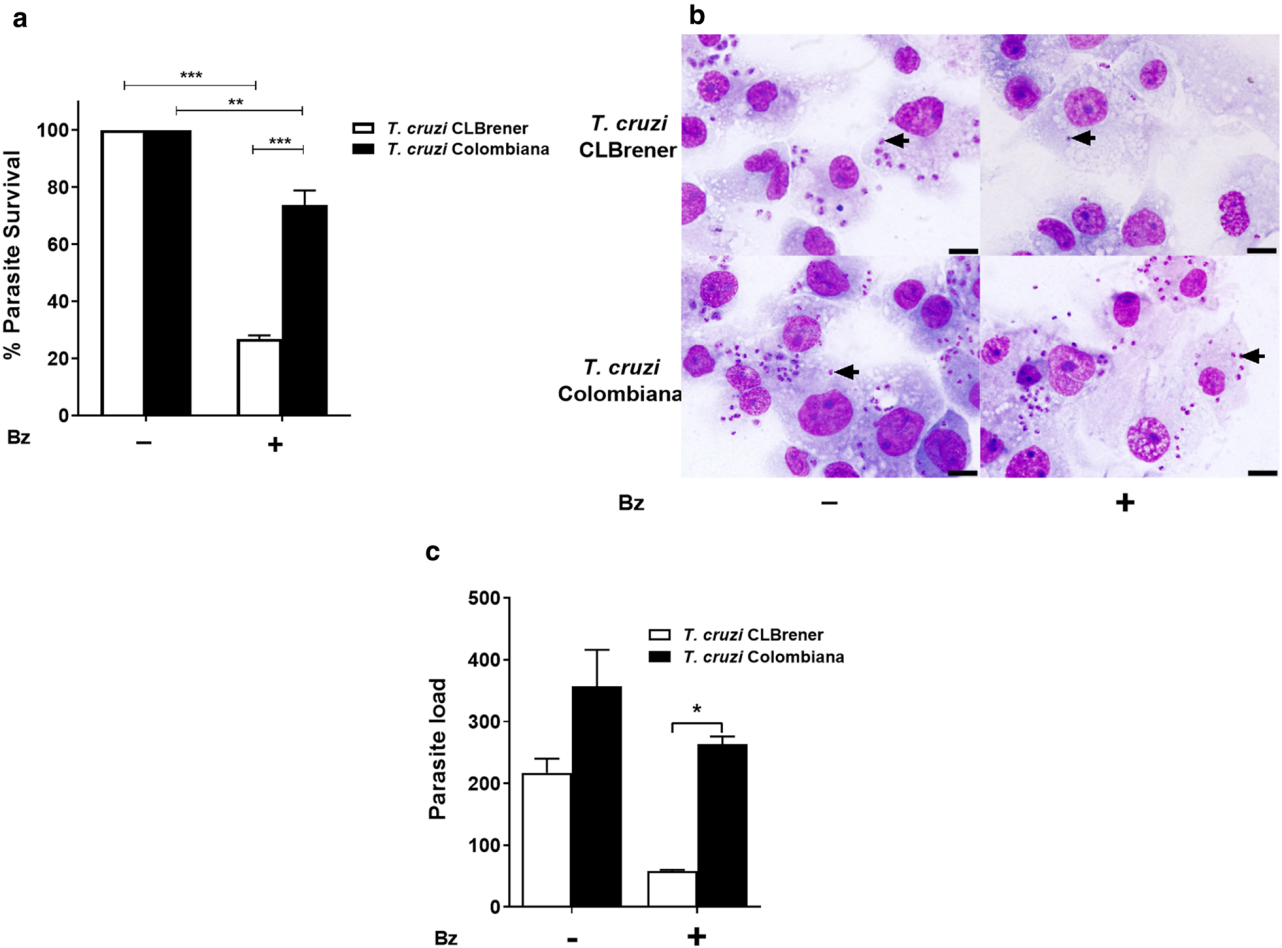
Effects of benznidazole on the intracellular viability of BZ-sensitive and BZ-resistant *T. cruzi* strains in human THP-1 MΦ cells. **a** Intracellular survival of CL Brener (sensitive) and Colombiana (resistant) *T. cruzi* strain amastigotes treated with BZ (13 µM). **b** Photomicrographs of Giemsa-stained THP-1 MФ cells treated with 13 µM BZ containing amastigotes of sensitive and resistant *T. cruzi* strains (arrow). **c** Parasitic load in the different groups described below. The results represent the average of two independent experiments + SEM. Significant differences were determined by two-way ANOVA, followed by Bonferroni’s multiple comparison test, *P < 0.05, **P < 0.01, and ***P < 0.001. *Scale bars*: 20 μm

The dose-response assays to assess the efficacy of the drug against intracellular amastigotes showed that the IC_50_ of BZ for the *T. cruzi* Colombiana strain was 2.82-fold greater than that for the CL Brener strain (Table 1). As expected, the percentage of intracellular parasite survival was significantly greater for the naturally resistant strain (Colombiana; 73.7%) in contrast to the sensitive CL Brener strain (26.7%) after treatment with 13 µM BZ (Fig. 1a). Differences in intracellular parasite survival for both *T. cruzi* strains after treatment with BZ in other host cell lines has also previously been reported [15, 22]. Although THP-1 MΦ cells infected with the Colombiana strain showed a higher parasite load than those infected with CL Brener after treatment with 13 µM BZ (ANOVA: *F*_(1, 2)_ = 84.0, *P* = 0.0117), no differences were observed for the number of amastigotes/cell in infected cells without treatment (Fig. 1b, c).

**Table 1:** The effect of benznidazole treatment on intracellular amastigotes of sensitive (CL Brener) and resistant (Colombiana) *Trypanosoma cruzi* strains in THP-1 macrophages

*T. cruzi* strain	IC_50_ (95% CI) BZ (μM)	Resistance index^a^
CL Brener	3.2 (2.47–4.12)	na
Colombiana	9.01 (5.67–14.32)	2.82

^a^Resistance index was calculated by the ratio of the IC_50_ value for the *T. cruzi* naturally benznidazole-resistant (Colombiana) and the sensitive (CL Brener) strains

Abbreviation: na, not applicable

### Effect of benznidazole treatment on drug transporter and oxidative stress gene expression levels in human macrophages infected with benznidazole-sensitive and naturally benznidazole-resistant *T. cruzi* strains

**Fig. 2:**
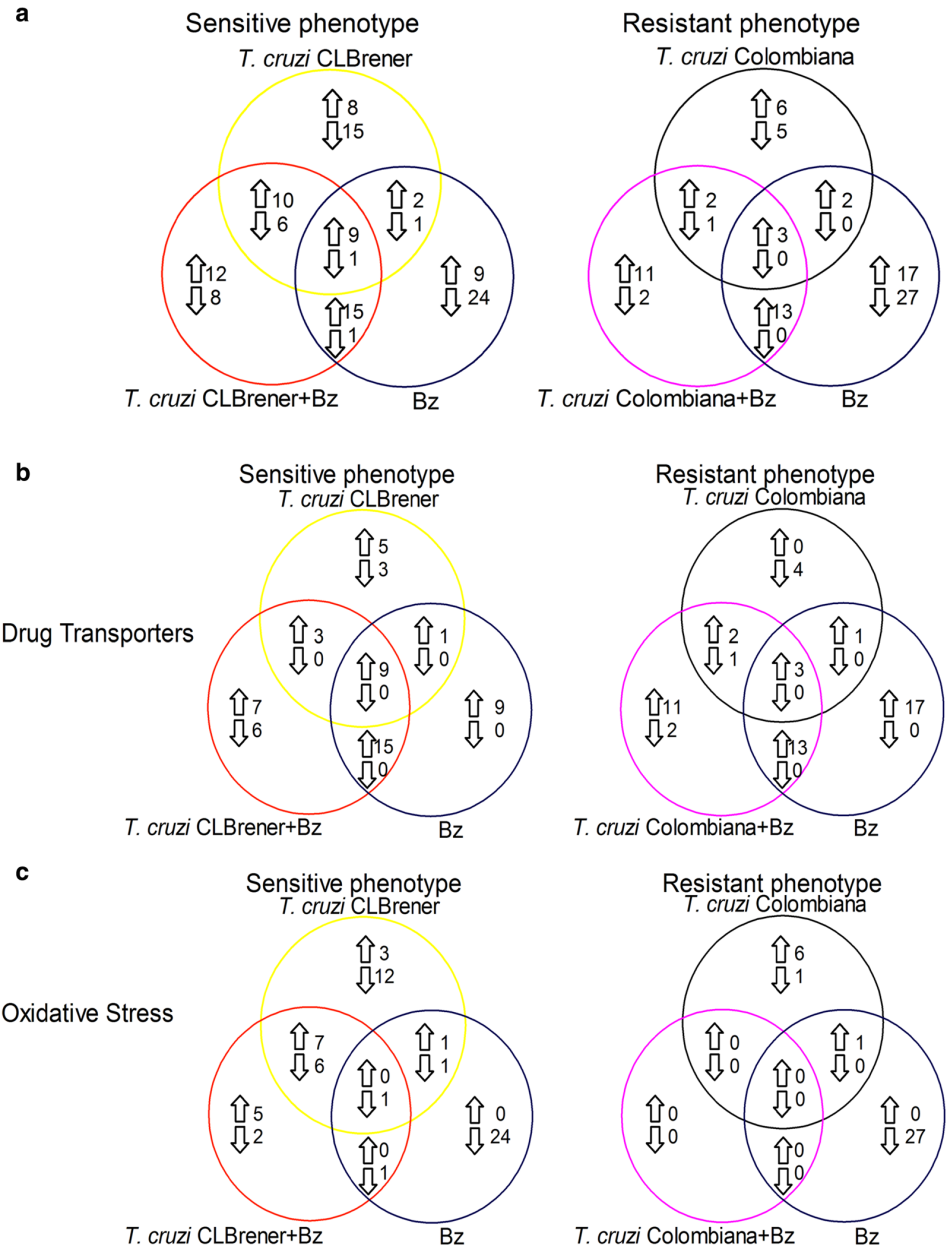
Venn diagram showing the number of transcripts upregulated and downregulated in human THP-1 MΦ cells. **a** Total number of commonly or differentially expressed gene transcripts in differentiated THP-1 macrophages. **b** Number of gene transcripts of the drug transporter pathway. **c** Number of gene transcripts of the oxidative stress pathway. THP-1 MΦ cells infected with *Trypanosoma cruzi* CL Brener strain (yellow circles), THP-1 MΦ cells treated with benznidazole (dark blue circles), THP-1 MΦ cells infected with *T. cruzi* CL Brener strain and then treated with benznidazole (red circles), THP-1 MΦ cells infected with *T. cruzi* Colombiana strain (black circles), or THP-1 MΦ cells infected with *T. cruzi* Colombiana strain and then treated with benznidazole (pink circles). Upregulated genes are indicated by up arrows, and downregulated genes are indicated by down arrows in the different groups. The number of gene transcripts in the THP-1 MΦ cells corresponds to the mean of three independent experiments

The determination of a possible association between parasite sensitivity phenotypes and their capacity to regulate human macrophage responses requires the use of naturally resistant pathogens to drugs [23]. We used the *T. cruzi*-macrophage-drug interaction model to address this assumption and to obtain global insights to understand the role of human responses to infection and drug treatment interaction by evaluating the gene expression profiles in human THP-1 MФ cells infected with CL Brener and Colombiana treated or not treated with BZ. Our results led to the identification of differentially regulated species-specific host genes and enabled us to identify potential markers associated with BZ resistance in human THP-1 macrophages infected with *T. cruzi*. A 3-fold difference in gene expression compared to the controls (THP-1 macrophages without infection and without treatment) was established as an arbitrary cut-off. Overall, the expression level of 62 (36.9%) of the evaluated genes changed in the host by at least 3-fold upon infection with CL Brener and BZ treatment, with 27.4% (46/168) of the genes being upregulated and 9.52% (16/168) of the genes being downregulated (Fig. 2a). In addition, the expression level of 32 (19.05%) genes changed in THP-1 MФ cells infected with Colombiana and treated with BZ, with 17.3% (29/168) of the genes being upregulated and 1.79% (3/168) of the genes being downregulated (Fig. 2a). Of the 46 upregulated genes in THP-1 MФ cells infected with CL Brener and treated with BZ, 73.9% and 26.1% belonged to the drug transporter and oxidative stress pathways, respectively (Fig. 2b, c). In contrast, all 29 genes upregulated in THP-1 MФ cells infected with Colombiana and treated with BZ belonged to the drug transporter group, and none belonged to the oxidative stress pathway (Fig. 2b).

**Fig. 3:**
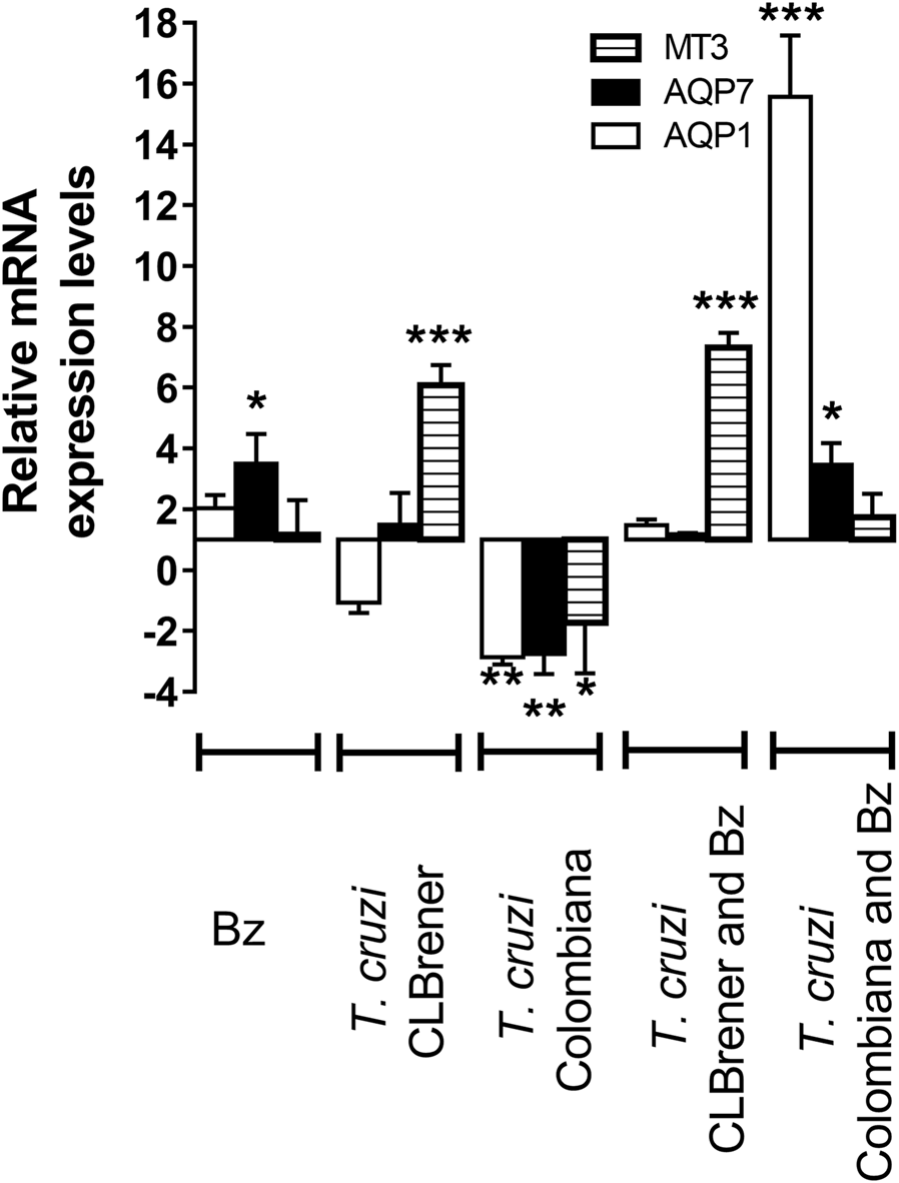
RT-qPCR validation of transcript expression of drug transporters and oxidative stress arrays in BZ-sensitive and BZ-resistant *T cruzi*-infected THP-1 MΦ cells treated with BZ. Relative mRNA expression is expressed as a ratio of transcripts in the study groups compared to transcripts in the control group (differentiated THP-1 macrophages without infection and without treatment). The values presented for the quantitative reverse-transcriptase real-time PCR analyses are the mean of the ratios of macrophage transcripts from two independent experiments. The gene expression in the study groups was normalized to *GAPDH*. The genes listed are *MT3*, metallothionein 3; *AQP1*, aquaporin 1; and *AQP7*, aquaporin 7. Significant differences were determined by two-way ANOVA, followed by Bonferroni’s multiple comparison test, *P < 0.05, **P < 0.01, and ***P < 0.001

In the *T. cruzi*-macrophage-BZ interaction model, we found changes in gene expression levels in pathways related to antioxidant defence, superoxide metabolism, oxidative stress response and solute drug transporters. Among the genes that were differentially expressed by infection and treatment, we focussed on the genes *AQP1*, *AQP7*, *AQP9* and *ABCB1* (*MDR1*), which could be involved in BZ uptake and drug activity against intracellular amastigotes (Fig. 3, Additional file 1: Table S1, Additional file 2: Table S2). Interestingly, no association data between *AQP* or *ABCB1* modulation has been demonstrated in *T. cruzi* [24]. However, the involvement of these proteins in drug uptake mechanisms by the host cell and the association with resistance phenotypes to BZ constitutes a broad field to be investigated. Furthermore, functional studies in other host cell response models have shown that the heterologous expression of the mammalian *AQP7* and *AQP9* genes allows for the absorption of AsIII and SbIII when expressed in *S. cerevisiae* or in *Xenopus laevis* oocytes [25]. Additionally, downregulation of the *AQP1* gene, which is homologous to the mammalian *AQP9* gene, has been reported to be associated with resistance phenotypes in *Leishmania* spp. [26, 27]. The regulation of human drug transporter and oxidative stress genes in response to *T. cruzi* infection and BZ treatment, regardless of the strain’s phenotype, has not been described to date in human macrophages to the best of our knowledge. These findings suggest that genes associated with drug transporters could be involved in the toxic effects of BZ in host cells.

THP-1 cells infected with *T. cruzi* and THP-1 MΦ cells treated with BZ showed a gene modulation profile that was different from that of infected and treated cells, particularly for genes involved in antioxidant defence (Additional file 2: Table S2). In the THP-1 MΦ cells infected with CL Brener, 17.3% of the genes (29/168) were upregulated, and three of these genes were modulated exclusively in this group (Fig. 2a, Additional file 1: Table S1, Additional file 2: Table S2). In the THP-1 MΦ cells infected with Colombiana, 13 upregulated genes were found, but none of these genes were exclusive to this group (Fig. 2a). In the THP-1 MΦ cells not infected but treated with BZ, 20.8% of the genes (35/168 genes) were upregulated, with three of the genes being exclusive to the group (Fig. 2a, Additional file 1: Table S1, Additional file 2: Table S2). Among the 35 genes that were upregulated in the treated group, we highlighted the expression levels of *ABCC2/MRP2* [ATP-binding cassette, sub-family C (CFTR/MRP), member 2]. Despite the high mRNA expression levels of *MRP2* observed in all treated conditions, the results suggest that this transporter may play a role in BZ detoxification. *MRP2* has been reported to be involved in xenobiotic efflux, which potentially induces oxidative stress in host cells [28]. Moreover, *MRP2* extrudes GSSG, which, along with *de novo* GSH synthesis, contributes to the return of normal GSH levels and redox homeostasis [28].

In the THP-1 MΦ cells infected with CL Brener, 13.7% of the genes (23/168 genes) were downregulated, with 13 of the genes belonged exclusively to this group (Fig. 2a, Additional file 1: Table S1, Additional file 2: Table S2). Among these 13 genes, we observed the modulation of *SOD1* (superoxide dismutase 1, soluble), *SOD2* (superoxide dismutase 2, mitochondrial), *TXN* (thioredoxin) and *TXNRD1* (thioredoxin reductase 1), which are related to antioxidant defence, and this finding is consistent with the ability of macrophages to control infection. In the THP-1 MΦ cells infected with Colombiana, six downregulated genes were found, one of which belonged solely to this group (Fig. 2a). Furthermore, it has been reported that the human response to *T. cruzi* infection has large differences that reflect the parasite’s adaptation to distinct environments during the infection of mammalian cells, including changes in energy sources, oxidative stress responses, cell cycle control and cell surface components [29]. Taken together, our results suggest that *T. cruzi* could modulate particular genes for its own protection and replication in the intracellular host environment.

In the THP-1 MΦ cells treated with BZ, 16.1% of genes (27/168 genes) were downregulated, with 14 genes belonging exclusively to this group (Fig. 2a, Additional file 1: Table S1, Additional file 2: Table S2). Among these genes, we identified *CAT* (catalase), *GCLC* (glutamate-cysteine ligase catalytic subunit), *GCLM* (glutamate-cysteine ligase modifier subunit), *GPX1* (glutathione peroxidase 1), *GSR* (glutathione reductase), *GSS* (glutathione synthetase) and *GSTP1* (glutathione S-transferase π1) (Additional file 2: Table S2). In contrast to our findings of downregulation of these genes, recent studies have shown enzymatic upregulation of CAT, SOD and GPX as a natural response to attenuate damage mediated by ROS/RNS after 12 h of exposure to BZ [28, 30]. These differences suggest that to evaluate the significance of these results, a strategy combining different time points could elucidate the true involvement of these molecules in host cell responses to BZ treatment.

Similar to antimony compounds, the first-line drugs for leishmaniasis treatment, the mode of action of BZ remains unclear, and the participation of the host cell in these mechanisms has been virtually unexplored [31]. Recently, it has been reported that BZ activity in *T. cruzi* is associated with the intracellular formation of BZ conjugates with thiols, producing an endogenous depletion of these molecules in the parasite, further increasing its defence against oxidative stress and generated electrophilic metabolites [6, 32].

BZ has been found to induce the overexpression and increase the activity of biotransformation enzymes (also referred to as drug metabolizing enzymes), such as GSTP (glutathione S-transferase π1), or drug transporters, such as P-gp and MRP2, in a dose-dependent manner in the HepG2 cell line [31]. Modulation of these enzymes was observed when cells were exposed to a concentration of 200 µM BZ, but not at lower doses [31]. Recently, in *T. cruzi*, it has been reported that high levels of gene expression and activity of the *MDR1* efflux transporter induce parasite resistance to BZ [33]. In 2016, Perdomo et al. [13] reported that P-gp and MRP2 activities were correlated with increased protein levels, inducing lower BZ intracellular accumulation in THP-1 human macrophages. Functional data from the inhibition of the expression of GSTP enzymes by PXR (pregnane X receptor) suggest that GSTP, P-gp and MRP2 are involved in BZ detoxification [31]. These results have subsequently been confirmed with *in vivo* studies in mice, where treatment with BZ increased the expression and activity of GSTP, P-gp and MRP2, mainly in the liver and in the proximal intestine [14]. The lack of *GSTP1* modulation in the *T. cruzi*-BZ-macrophage interaction model is consistent with previous reports, probably due to the working dose of 13 µM, which is consistent with the absence of modulation in low-dose exposure to BZ previously reported by Rigalli et al. [31]. To the best of our knowledge, no studies in the THP-1 human cell line have found an association between resistance phenotypes and drug sensitivity in intracellular *T. cruzi* strain interactions or described their effects on the modulation of gene expression in host cells after drug treatment. However, several studies have focused on parasite resistance to BZ, employing methods such as proteomics and metabolomics, or a host cell response to *T. cruzi* infection alone [29, 32, 34–39].

## Conclusions

Our findings support the value of gene expression studies focused on the host response to pathogens and drug treatments integrated with functional and metabolic data to identify potentially novel targets for the treatment of this important and neglected tropical disease.

## Additional files

**Additional file 1: Table S1.** Regulation of drug transporter gene expression levels in human THP-1 MΦ cells by benznidazole (BZ) and *T. cruzi* infection.

**Additional file 2: Table S2.** Regulation of oxidative stress gene expression levels in human THP-1 MΦ cells by benznidazole (BZ) and *T. cruzi* infection.

## Abbreviations

NFX: nifurtimox
BZ: benznidazole
qPCR: quantitative polymerase chain reaction
RT-qPCR: real-time quantitative PCR
THP-1 MΦ: human acute monocytic leukaemia cell line differentiated into macrophages
