# Supplementary material for: Drug transporter and oxidative stress gene expression in human macrophages infected with benznidazole-sensitive and naturally benznidazole-resistant Trypanosoma cruzi parasites treated with benznidazole
Source: Parasit Vectors. 2019 May 24;12:262. doi: 10.1186/s13071-019-3485-9 (PMC6534881; doi:10.1186/s13071-019-3485-9)
Supplement: Supplementary file 1 — Additional file 1: Table S1. Regulation of drug transporter gene expression levels in human THP-1 MΦ cells by benznidazole (BZ) and T. cruzi infection. [file 13071_2019_3485_MOESM1_ESM.pdf]

Table S1. Regulation of drug transporter gene expression levels in Human THP-1 MΦ cells by benznidazole (BZ) and *T. cruzi* infection

Refseq	Symbol	Description Drug Transporters	Fold change gene expression (95% CI) [a]				
			BZ	*T. cruzi* CLBrener	*T. cruzi* Colombiana	*T. cruzi* CLBrener and BZ	*T. cruzi* Colombiana and BZ
NM_005502	*ABCA1*	ATP-binding cassette, sub-family A (ABC1), member 1	8.8356 (8.08, 9.60)	1.0586 (0.17, 1.95)	1.9537 (1.32, 2.59)	-1.3745 (-2.01, -0.74)	5.0445 (4.16, 5.93)
NM_173076	*ABCA12*	ATP-binding cassette, sub-family A (ABC1), member 12	3.3908 (3.11, 3.68)	-3.3845 (-4.31,-2.46)	19.1207 (17.3, 20.9)	-1.4934 (-1.69, -1.30)	1.826 (1.23, 2.42)
NM_152701	*ABCA13*	ATP-binding cassette, sub-family A (ABC1), member 13	3.665 (2.91, 4.42)	-1.6538 (-2.09, -1.21)	1.8195 (1.09, 2.55)	-1.5753 (-2.12, -1.03)	2.3703 (2.04, 2.70)
NM_001606	*ABCA2*	ATP-binding cassette, sub-family A (ABC1), member 2	2.7644 (2.35, 3.17)	-2.0921 (-2.10, -2.09)	-1.5045 (-1.08, -1.93)	1.4402 (1.37, 1.51)	2.2698 (2.26, 2.28)
NM_001089	*ABCA3*	ATP-binding cassette, sub-family A (ABC1), member 3	2.895 (2.25, 3.54)	-1.1584 (-1.38, -0.94)	1.4015 (0.77, 2.04)	2.2194 (1.20, 3.24)	2.1489 (1.93, 2.37)
NM_000350	*ABCA4*	ATP-binding cassette, sub-family A (ABC1), member 4	14.9455 (14.2, 15.7)	1.4542 (1.06, 1.85)	1.5065 (1.27, 1.75)	3.5198 (3.50, 3.54)	5.3066 (4.71, 5.91)
NM_018672	*ABCA5*	ATP-binding cassette, sub-family A (ABC1), member 5	3.0866 (2.54, 3.63)	-1.2496 (-1.31, -1.18)	1.544 (0.85, 2.24)	3.4305 (2.80, 4.06)	1.6613 (1.46, 1.87)
NM_080283	*ABCA9*	ATP-binding cassette, sub-family A (ABC1), member 9	5.2366 (4.82, 5.66)	1.3701 (1.04, 1.70)	1.1433 (0.97, 1.32)	3.7423 (2.97, 4.52)	1.7544 (1.39, 2.12)
NM_000927	*ABCB1/PgP*	ATP-binding cassette, sub-family B (MDR/TAP), member 1	1.8861 (1.21, 2.57)	1.1925 (1.07, 1.31)	1.1721 (0.61, 1.74)	3.2343 (3.22, 3.25)	2.0662 (1.45, 2.69)
NM_003742	*ABCB11/BSEP*	ATP-binding cassette, sub-family B (MDR/TAP), member 11	2.134 (1.37, 2.90)	-1.3246 (-1.68, -0.97)	-1.2274 (-1.72, -0.73)	1.5261 (1.28, 1.78)	1.4648 (1.15, 1.78)
NM_000443	*ABCB4*	ATP-binding cassette, sub-family B (MDR/TAP), member 4	2.6217 (2.46, 2.78)	-1.5689 (-2.25, -0.89)	1.2873 (0.99, 1.58)	3.0113 (2.94, 3.08)	3.0951 (2.03, 4.16)
NM_178559	*ABCB5*	ATP-binding cassette, sub-family B (MDR/TAP), member 5	1.251 (0.38, 2.13)	-5.7229 (-6.27, -5.18)	-2.5825 (-3.70, -1.46)	-2.927 (-3.20, -2.66)	-1.3283 (-1.59, -1.06)
NM_005689	*ABCB6*	ATP-binding cassette, sub-family B (MDR/TAP), member 6	4.7989 (4.28, 5.32)	-1.855 (-2.32, -1.39)	1.4148 (1.29, 1.54)	1.3542 (0.69, 2.02)	1.7546 (1.24, 2.27)
NM_004996	*ABCC1*	ATP-binding cassette, sub-family C (CFTR/MRP), member 1	2.1807 (1.71, 2.65)	-1.7733 (-2.00, -1.55)	1.6135 (0.87, 2.36)	-1.2376 (-2.30, -0.17)	2.6878 (2.30, 3.07)
NM_033450	*ABCC10*	ATP-binding cassette, sub-family C (CFTR/MRP), member 10	3.1364 (3.08, 3.19)	2.5369 (2.05, 3.02)	4.8422 (4.66, 5.03)	2.7291 (2.65, 2.80)	5.8578 (5.35, 6.37)
NM_032583	*ABCC11*	ATP-binding cassette, sub-family C (CFTR/MRP), member 11	4.2552 (4.20, 4.31)	1.7608 (0.99, 2.53)	1.1439 (1.13, 1.15)	2.1467 (2.05, 2.24)	5.7217 (5.10, 6.35)

NM_033226	*ABCC12*	ATP-binding cassette, sub-family C (CFTR/MRP), member 12	3.056 (1.93, 4.18)	3.554 (2.55, 4.56)	1.7714 (1.76, 1.78)	3.3244 (2.40, 4.24)	2.6057 (1.83, 3.38)
NM_000392	*ABCC2*	ATP-binding cassette, sub-family C (CFTR/MRP), member 2	3.019 (2.41, 3.63)	1.1675 (0.96, 1.38)	1.109 (0.84, 1.38)	3.6541 (3.27, 4.03)	3.5141 (3.19, 3.83)
NM_003786	*ABCC3*	ATP-binding cassette, sub-family C (CFTR/MRP), member 3	1.9902 (1.51, 2.47)	-1.1719 (-1.55, -0.79)	1.419 (0.88, 1.95)	2.8434 (2.01, 3.67)	2.4694 (1.98, 2.96)
NM_005845	*ABCC4*	ATP-binding cassette, sub-family C (CFTR/MRP), member 4	1.7642 (1.21, 2.32)	-2.2396 (-2.33, -2.15)	-1.0433 (-1.22, -0.87)	1.8348 (1.58, 2.09)	1.3854 (0.75, 2.02)
NM_005688	*ABCC5*	ATP-binding cassette, sub-family C (CFTR/MRP), member 5	1.8629 (1.51, 2.21)	1.2937 (0.53, 2.06)	-1.2903 (-1.68, -0.90)	2.5963 (2.39, 2.80)	1.8851 (0.90, 2.87)
NM_000033	*ABCD1*	ATP-binding cassette, sub-family D (ALD), member 1	1.427 (0.74, 2.11)	3.2834 (2.70, 3.87)	2.0004 (1.95, 2.05)	14.1085 (13.8, 14.5)	6.9668 (6.53, 7.40)
NM_002858	*ABCD3*	ATP-binding cassette, sub-family D (ALD), member 3	1.9167 (1.37, 2.46)	-1.5482 (-1.74, -135)	-1.158 (-1.18, -1.14)	2.4303 (2.31, 2.55)	2.018 (1.32, 2.71)
NM_005050	*ABCD4*	ATP-binding cassette, sub-family D (ALD), member 4	2.6129 (2.32, 2.91)	1.1091 (0.66, 1.56)	-5.3489 (-6.70, -4.00)	-2.677 (-4.27, -1,08)	3.1736 (2.27, 4.08)
NM_001090	*ABCF1*	ATP-binding cassette, sub-family F (GCN20), member 1	3.3575 (2.56, 4.16)	-4.1034 (-5.24, -2.97)	-1.0434 (-2.07, -0.01	-2.3476 (-3.64, -1.06)	3.1305 (2.53, 3.73)
NM_004827	*ABCG2/BCRP*	ATP-binding cassette, sub-family G (WHITE), member 2	2.7632 (2.62, 2.91)	-2.9836 (-3.73, -2.23)	1.3562 (0.75, 1.96)	-1.8374 (-2.25, -1.42)	2.2007 (1.88, 2.52)
NM_022437	*ABCG8*	ATP-binding cassette, sub-family G (WHITE), member 8	2.1329 (1.41, 2.85)	1.4365 (1.25, 1.62)	2.8977 (2.49, 3.31)	2.483 (2.01, 2.95)	2.3558 (2.21, 2.50)
NM_198098	*AQP1*	Aquaporin 1 (Colton blood group)	2.0748 (1.4, 2.75)	-1.1081 (-1.73, -0.49)	-1.3257 (-1.40, -1.25)	-1.0878 (-1.82, -0.36)	17.6716 (16.20, 19.14)
NM_001170	*AQP7*	Aquaporin 7	3.5591 (3.52, 3.60)	1.4315 (0.001, 2.93)	-1.2766 (-1.75, -0.81)	-1.3451 (-1.39, -1.30)	3.9364 (3.28, 4.59)
NM_020980	*AQP9*	Aquaporin 9	2.1621 (1.58, 2.74)	1.4487 (1.02, 1.88)	-1.0944 (-1.38, -0.81)	3.2835 (2.40, 4.17)	1.948 (1.74, 2.15)
NM_001694	*ATP6V0C*	ATPase, H+ transporting, lysosomal 16kDa, V0 subunit c	2.1872 (1.93, 2.45)	-1.3408 (-1.72, -0.96)	1.5513 (1.52, 1.58)	3.4037 (2.57, 4.23)	2.5567 (1.95, 3.16)
NM_000052	*ATP7A*	ATPase, Cu++ transporting, alpha polypeptide	1.7627 (1.39, 2.14)	-1.0946 (-1.22, -0.97)	1.1958 (0.60, 1.79)	3.3945 (2.92, 3.87)	2.1636 (1.03, 3.30)
NM_000053	*ATP7B*	ATPase, Cu++ transporting, beta polypeptide	1.623 (1.50, 1.74)	3.7543 (3.44, 4.07)	-1.0794 (-1.50, -0.66)	2.907 (2.59, 3.23)	2.5298 (1.44, 3.62)
NM_017458	*MVP*	Major vault protein	-2.7364 (-4.4, -1.03)	5.8542 (4.77, 6.94)	6.354 (5.55, 7.16)	12.5729 (12.5, 12.7)	7.9245 (6.99, 8.86)
NM_003049	*SLC10A1*	Solute carrier family 10 (sodium/bile acid cotransporter family), member 1	2.3528 (1.05, 3.65)	1.4134 (0.001, 2.88)	1.9783 (1.59, 2.36)	2.7241 (2.01, 3.44)	6.0752 (5.09, 7.06)
NM_000452	*SLC10A2*	Solute carrier family 10 (sodium/bile acid cotransporter family), member 2	1.1758 (0.05, 2.30)	2.4387 (1.03, 3.85)	-1.4672 (-1.48, 1.46)	1.2791 (1.23, 1.33)	1.0026 (0.22, 1.78)

NM_005073	*SLC15A1*	Solute carrier family 15 (oligopeptide transporter), member 1	3.056 (1.93, 4.18)	1.6887 (1.44, 1.94)	2.9937 (1.91, 4.07)	3.3244 (3.27, 3.38)	2.6057 (1.83, 3.38)
NM_021082	*SLC15A2*	Solute carrier family 15 (H+/peptide transporter), member 2	2.3588 (1.82, 2.90)	-2.3112 (-3.02, -1.60)	-1.5072 (-1.51, -1.50)	-2.6217 (-3.45, -1.80)	1.2813 (1.09, 1.47)
NM_003051	*SLC16A1*	Solute carrier family 16, member 1 (monocarboxylic acid transporter 1)	3.1213 (2.75, 3.49)	1.3007 (0.85, 1.75)	1.7263 (0.96, 2.46)	-1.0842 (-1.38, -0.79)	2.656 (2.51, 2.80)
NM_006517	*SLC16A2*	Solute carrier family 16, member 2 (monocarboxylic acid transporter 8)	2.566 (2.21, 2.92)	-2.5086 (-3.04, -1.98)	-3.4774 (-3.68, -3.27)	-2.9677 (-3.01, -2.93)	1.6738 (1.03, 2.31)
NM_004207	*SLC16A3*	Solute carrier family 16, member 3 (monocarboxylic acid transporter 4)	1.0271 (0.001, 2.14)	2.2083 (2.04, 2.38)	-1.4942 (-1.73, -1.26)	1.7427 (0.83, 2.66)	3.6023 (3.31, 3.89)
NM_194255	*SLC19A1*	Solute carrier family 19 (folate transporter), member 1	2.0732 (1.46, 2.69)	1.4654 (1.00, 1.93)	-1.2613 (-1.36, -1.16)	1.2391 (1.01, 1.46)	1.7391 (1.16, 2.32)
NM_006996	*SLC19A2*	Solute carrier family 19 (thiamine transporter), member 2	8.3766 (7.99, 8.76)	4.7097 (4.53, 4.89)	1.2852 (0.88, 1.69)	5.5067 (4.83, 6.19)	6.8512 (6.51, 7.20)
NM_025243	*SLC19A3*	Solute carrier family 19, member 3	3.056 (1.93, 4.18)	2.0108 (1.85, 2.17)	1.7714 (1.76, 1.78)	3.3244 (2.40, 4.24)	2.6057 (1.83, 3.38)
NM_003057	*SLC22A1/ OCT 1*	Solute carrier family 22 (organic cation transporter), member 1	1.8762 (1.85, 1.90)	1.195 (1.08, 1.31)	-1.2168 (-1.37, -1.06)	3.4469 (3.04, 3.85)	2.7604 (1.02, 4.50)
NM_003058	*SLC22A2/ OCT2*	Solute carrier family 22 (organic cation transporter), member 2	3.056 (1.93, 4.18)	1.7672 (1.47, 2.07)	1.7714 (1.76, 1.78)	3.3244 (2.40, 4.24)	2.6057 (1.83, 3.38)
NM_021977	*SLC22A3*	Solute carrier family 22 (extraneuronal monoamine transporter), member 3	3.056 (1.93, 4.18)	2.6619 (1.92, 3.40)	1.7714 (1.76, 1.78)	3.3244 (2.40, 4.24)	2.6057 (1.83, 3.38)
NM_004790	*SLC22A6*	Solute carrier family 22 (organic anion transporter), member 6	3.056 (1.93, 4.18)	3.4682 (2.48, 4.46)	1.7714 (1.76, 1.78)	4.0244 (3.18, 4.87)	2.6057 (1.83, 3.38)
NM_006672	*SLC22A7*	Solute carrier family 22 (organic anion transporter), member 7	3.8522 (3.75, 3.96)	1.6887 (1.44, 1.94)	1.7714 (1.76, 1.78)	3.3244 (2.40, 4.24)	2.6057 (1.83, 3.38)
NM_004254	*SLC22A8*	Solute carrier family 22 (organic anion transporter), member 8	3.056 (1.93, 4.18)	1.6887 (1.44, 1.94)	1.7714 (1.76, 1.78)	3.3244 (2.40, 4.24)	2.6057 (1.83, 3.38)
NM_080866	*SLC22A9*	Solute carrier family 22 (organic anion transporter), member 9	3.056 (1.93, 4.18)	1.6887 (1.44, 1.94)	1.8034 (1.77, 1.84)	3.3244 (2.40, 4.24)	2.6057 (1.83, 3.38)
NM_014251	*SLC25A13*	Solute carrier family 25, member 13 (citrin)	2.2752 (1.94, 2.61)	1.9146 (1.73, 2.10)	1.2653 (0.74, 1.79)	1.302 (0.84, 1.77)	1.9758 (1.44, 2.51)
NM_004213	*SLC28A1*	Solute carrier family 28 (sodium-coupled nucleoside transporter), member 1	3.3007 (3.02, 3.58)	3.7505 (3.06, 4.44)	2.3247 (1.98, 2.67)	2.0673 (1.66, 2.48)	3.6234 (3.01, 4.24)
NM_004212	*SLC28A2*	Solute carrier family 28 (sodium-coupled nucleoside transporter), member 2	3.9949 (3.97, 4.02)	4.4219 (4.42, 4.43)	2.1506 (1.74, 2.56)	3.1402 (2.66, 3.62)	6.092 (5.65, 6.53)
NM_022127	*SLC28A3*	Solute carrier family 28 (sodium-coupled nucleoside transporter), member 3	15.0451 (14.3, 15.8)	3.5086 (3.31, 3.70)	2.7257 (2.37, 3.08)	6.1112 (5.44, 6.79)	5.3706 (4.08, 6.66)
NM_004955	*SLC29A1*	Solute carrier family 29 (nucleoside transporters), member 1	1.3861 (0.79, 1.98)	1.9033 (1.56, 2.25)	-1.4167 (-1.45, -1.39)	1.876 (1.68, 2.07)	2.4822 (2.32, 2.65)
NM_001532	*SLC29A2*	Solute carrier family 29 (nucleoside	4.5503	3.7463	1.3205	3.8454	3.132

		transporters), member 2	(4.54, 4.56)	(3.44, 4.05)	(1.17, 1.47)	(3.70, 3.99)	(2.31, 3.95)
NM_006516	*SLC2A1*	Solute carrier family 2 (facilitated glucose transporter), member 1	1.3604 (1.01, 1.71)	2.7508 (2.73, 2.77)	-1.549 (-1.69, -1.41)	3.3322 (2.77, 3.89)	2.7975 (2.00, 3.60)
NM_000340	*SLC2A2*	Solute carrier family 2 (facilitated glucose transporter), member 2	1.269 (0.72, 1.82)	-1.2675 (-2.33, -0.21)	-1.4126 (-2.46, -0.36)	1.1492 (1.03, 1.26)	-1.021 (-1.16, -0.88)
NM_006931	*SLC2A3*	Solute carrier family 2 (facilitated glucose transporter), member 3	-1.2186 (-2.32, -0.12)	2.1591 (1.27, 3.05)	-1.4209 (-1.62, -1.22)	-3.4331 (-4.38, -2.49)	-1.1388 (-2.00, -0.28)
NM_001859	*SLC31A1*	Solute carrier family 31 (copper transporters), member 1	-1.112 (-1.71, -0.51)	-1.0471 (-1.94, -0.16)	-4.79 (-4.89, -4.69)	-11.1948 (-12.5, -9.85)	3.2785 (3.18, 3.37)
NM_018976	*SLC38A2*	Solute carrier family 38, member 2	2.3826 (2.20, 2.57)	3.0159 (2.62, 3.42)	1.6274 (1.32, 1.93)	1.7504 (1.44, 2.06)	3.8786 (3.78, 3.98)
NM_033518	*SLC38A5*	Solute carrier family 38, member 5	2.1633 (1.26, 3.07)	6.3774 (6.35, 6.40)	4.6764 (3.28, 6.07)	2.9001 (2.01, 3.79)	6.1328 (5.12, 7.14)
NM_000341	*SLC3A1*	Solute carrier family 3 (cystine, dibasic and neutral amino acid transporters, activator of cystine, dibasic and neutral amino acid transport), member 1	2.2602 (1.28, 3.24)	2.4366 (2.03, 2.84)	1.5401 (0.84, 2.24)	1.2418 (0.75, 1.74)	1.7039 (1.65, 1.76)
NM_002394	*SLC3A2*	Solute carrier family 3 (activators of dibasic and neutral amino acid transport), member 2	-1.0041 (-1.36, -0.64)	5.1437 (5.07, 5.22)	2.1421 (1.40, 2.88)	2.832 (2.49, 3.18)	3.5839 (2.88, 4.28)
NM_000343	*SLC5A1*	Solute carrier family 5 (sodium/glucose cotransporter), member 1	6.3763 (5.79, 6.97)	3.0323 (2.72, 3.35)	2.7309 (2.50, 2.96)	6.1337 (7.11, 5.15)	13.6043 (13.4, 13.9)
NM_014227	*SLC5A4*	Solute carrier family 5 (low affinity glucose cotransporter), member 4	32.0479 (32.02, 32.08)	5.0713 (4.77, 5.38)	14.3267 (14.3, 14.4)	15.5081 (14.7, 16.3)	15.3738 (14.9, 15.8)
NM_014331	*SLC7A11*	Solute carrier family 7 (anionic amino acid transporter light chain, xc- system), member 11	-1.9055 (-1.91, -1.90)	-1.0149 (-1.06, -0.97)	-1.7866 (-2.13, -1.44)	-1.0402 (-1.29, -0.79)	1.379 (1.32, 1.44)
NM_003486	*SLC7A5*	Solute carrier family 7 (amino acid transporter light chain, L system), member 5	1.0911 (0.97, 1.21)	1.0439 (0.95, 1.14)	-1.4513 (-2.04, -0.86)	-1.0551 (-1.26, -0.85)	1.4836 (1.46, 1.51)
NM_003983	*SLC7A6*	Solute carrier family 7 (amino acid transporter light chain, y+L system), member 6	-1.025 (-1.38, -0.67)	1.3299 (1.11, 1.55)	-2.814 (-3.66, -1.97)	-1.0253 (-1.41, -0.64)	1.2307 (0.52, 1.94)
NM_003982	*SLC7A7*	Solute carrier family 7 (amino acid transporter light chain, y+L system), member 7	-1.7735 (-2.22, -1.33)	-1.1641 (-1.73, -0.59)	-18.8964 (-20.6, -17.2)	-17.0648 (-18.9, -15.3)	-7.7804 (-9.70, -5.86)
NM_182728	*SLC7A8*	Solute carrier family 7 (amino acid transporter light chain, L system), member 8	-2.7067 (-3.36, -2.05)	1.2989 (1.14, 1.46)	-2.8259 (-3.05, -2.60)	-6.5917 (-8.54, -4.64)	-8.4354 (-10.33, -6.54)
NM_014270	*SLC7A9*	Solute carrier family 7 (glycoprotein-associated amino acid transporter light chain, bo,+ system), member 9	3.056 (1.93, 4.18)	1.6887 (1.44, 1.94)	1.7714 (1.76, 1.78)	3.3244 (2.40, 4.24)	2.6057 (1.83, 3.38)
NM_021094	*SLCO1A2*	Solute carrier organic anion transporter family, member 1A2	3.9709 (3.95, 3.99)	2.947 (2.10, 3.79)	1.7714 (1.76, 1.78)	3.3244 (2.40, 4.24)	2.6057 (1.83, 3.38)

NM_006446	*SLCO1B1/ OATP1B1*	Solute carrier organic anion transporter family, member 1B1	3.4324 (2.29, 4.57)	2.062 (1.82, 2.30)	1.7714 (1.76, 1.78)	3.8811 (3.03, 4.73)	3.0144 (2.02, 4.01)
NM_019844	*SLCO1B3/ OATP1B3*	Solute carrier organic anion transporter family, member 1B3	3.056 (1.93, 4.18)	1.6887 (1.44, 1.94)	1.7943 (1.77, 1.82)	3.3244 (2.40, 4.24)	2.6057 (1.83, 3.38)
NM_005630	*SLCO2A1*	Solute carrier organic anion transporter family, member 2A1	3.9631 (3.96, 3.97)	3.6653 (3.39, 3.94)	4.8951 (4.61, 5.18)	5.9932 (5.26, 6.73)	4.0775 (3.31, 4.84)
NM_007256	*SLCO2B1*	Solute carrier organic anion transporter family, member 2B1	-1.1707 (-1.35, -0.99)	1.2463 (1.18, 1.31)	-1.2194 (-1.26, -1.18)	-1.3366 (-1.41, -1.26)	1.7312 (1.01, 2.45)
NM_013272	*SLCO3A1*	Solute carrier organic anion transporter family, member 3A1	1.42 (0.79, 2.05)	1.6121 (0.94, 2.28)	1.215 (0.65, 1.78)	1.9214 (1.35, 2.49)	2.2281 (1.97, 2.49)
NM_016354	*SLCO4A1*	Solute carrier organic anion transporter family, member 4A1	1.3292 (0.58, 2.08)	2.1667 (1.89, 2.44)	1.1936 (0.88, 1.51)	2.7124 (2.66, 2.76)	2.74 (2.37, 3.11)
NM_000593	*TAP1*	Transporter 1, ATP-binding cassette, sub-family B (MDR/TAP)	-1.8349 (-1.92, -1.75)	4.4555 (3.79, 5.12)	1.8309 (1.26, 2.40)	7.5054 (7.38, 7.63)	7.1565 (7.14, 7.17)
NM_000544	*TAP2*	Transporter 2, ATP-binding cassette, sub-family B (MDR/TAP)	-1.4977 (-1.60, -1.40)	3.6442 (3.30, 3.98)	-1.2403 (-1.39, -1.09)	2.8418 (2.45, 3.24)	3.2699 (2.57, 3.97)
NM_003374	*VDAC1*	Voltage-dependent anion channel 1	-1.5724 (-2.42, -0.72)	-1.5799 (-1.76, -1.40)	-3.7132 (-3.75, -3.67)	-24.0755 (-26.06, -22.09)	1.0031 (0.24, 1.76)
NM_003375	*VDAC2*	Voltage-dependent anion channel 2	-1.8089 (-2.31,-1.31)	-2.6632 (-3.50, -1.82)	-1.9369 (-2.02, -1.85)	-10.5245 (-12.16, -8.89)	-11.0348 (-12.9, -9.21)

[a] Regulation of gene expression was measured after 2 days of incubation with 13 µM BZ using the RT^2 Human Drug Transporters PCR expression array analysis (QIAGEN). Data are represented as fold change and 95 % confidence interval (95% CI)
