# Supplementary material for: Drug transporter and oxidative stress gene expression in human macrophages infected with benznidazole-sensitive and naturally benznidazole-resistant Trypanosoma cruzi parasites treated with benznidazole
Source: Parasit Vectors. 2019 May 24;12:262. doi: 10.1186/s13071-019-3485-9 (PMC6534881; doi:10.1186/s13071-019-3485-9)
Supplement: Supplementary file 2 — Additional file 2: Table S2. Regulation of oxidative stress gene expression levels in human THP-1 MΦ cells by benznidazole (BZ) and T. cruzi infection. [file 13071_2019_3485_MOESM2_ESM.pdf]

Table S2. Regulation of Oxidative Stress gene expression levels in Human THP-1 MΦ cells by benznidazole (BZ) and *T. cruzi* infection

			Fold change gene expression (95% CI) [a]				
Refseq	Symbol	Description Oxidative Stress	BZ	*T. cruzi* *CLBrener*	*T. cruzi* *Colombiana*	*T. cruzi* CLBrener and BZ	*T. cruzi* *Colombiana* and BZ
NM_000477	*ALB*	Albumin	-7.1566 (-7.26, -7.06)	3.8275 (2.89, 4.77)	3.089 (2.70, 3.48)	2.7396 (2.36, 3.12)	-1.3966 (-1.77, -1.02)
NM_000697	*ALOX12*	Arachidonate 12-lipoxygenase	-1.4136 (-2.32, -0.51)	2.2074 (1.90, 2.52)	4.2601 (3.79, 4.73)	1.9392 (1.80, 2.07)	1.1181 (0.89, 1.35)
NM_001159	*AOX1*	Aldehyde oxidase 1	-6.9266 (-8.26, -5.59)	1.4278 (1.22, 1.64)	-1.5476 (-1.91, -1.18)	-1.6045 (-1.84, -1.37)	-1.6952 (-1.91, -1.48)
NM_000041	*APOE*	Apolipoprotein E	-47.5847 (-49.1, -46.0)	1.9214 (0.77, 3.07)	6.4708 (6.41, 6.53)	-1.013 (-1.20, -0.83)	1.899 (1.61, 2.19)
NM_004045	*ATOX1*	ATX1 antioxidant protein 1 homolog (yeast)	-6.9257 (-7.88, -5.97)	3.3881 (2.84, 3.94)	2.3829 (2.02, 2.74)	5.9634 (5.17, 6.75)	2.9795 (2.71, 3.25)
NM_004052	*BNIP3*	BCL2/adenovirus E1B 19kDa interacting protein 3	-3.2217 (-3.89, -2.56)	2.5693 (1.96, 3.18)	2.0203 (1.84, 2.20)	2.4901 (2.15, 2.83)	-1.1698 (-1.22, -1-12)
NM_001752	*CAT*	Catalase	-9.2892 (-10.5, -8.05)	-1.3204 (-1.66, -0.98)	-1.0828 (-1.11, -1.06)	1.5945 (1.56, 1.63)	-2.0188 (-2.07, -1.97)
NM_002985	*CCL5*	Chemokine (C-C motif) ligand 5	-5.8697 (-6.77, -4.97)	27.0417 (26.6, 27.5)	1.9063 (1.57, 2.25)	41.2969 (41.1, 41.5)	1.9409 (1.87, 2.01)
NM_005125	*CCS*	Copper chaperone for superoxide dismutase	-7.7104 (-9.01, -6.41)	2.0433 (1.68, 2.41)	1.1272 (0.95, 1.30)	4.1296 (4.00, 4.26)	-1.0418 (-1.32, -0.77)
NM_000397	*CYBB*	Cytochrome b-245, beta polypeptide	-10.5767 (-11.7, -9.45)	-3.8593 (-3.95, -3.77)	-1.2467 (-1.36, -1.13)	-2.3447 (-2.56, -2.13)	-2.22 (-2.47,-1.97)
NM_134268	*CYGB*	Cytoglobin	-2.2301 (-3.50, -0.96)	2.7594 (2.38, 3.14)	-2.9181 (-2.95, -2.89)	2.1422 (1.77, 2.52)	-1.2172 (-1.30, -1.14)
NM_014762	*DHCR24*	24-dehydrocholesterol reductase	-12.5377 (-13.9, -11.2)	1.0898 (0.92, 1.26)	-1.6486 (-2.29, -1.01)	1.7008 (1.43, 1.97)	-1.9532 (-1.97, -1.93)
NM_175940	*DUOX1*	Dual oxidase 1	-1.5033 (-2.38, -0.62	1.7717 (1.72, 1.82)	2.9928 (2.76, 3.22)	2.4626 (1.94, 2.98)	-1.7554 (-1.82, -1.69)
NM_014080	*DUOX2*	Dual oxidase 2	8.1488 (7.01, 9.29)	3.3288 (3.12, 3.54)	8.9351 (8.27, 9.60)	2.5355 (2.34, 2.74)	1.4536 (0.87, 2.03)
NM_004417	*DUSP1*	Dual specificity phosphatase 1	-5.5259 (-6.28, -4.77)	4.6016 (4.06, 5.15)	-2.8959 (-3.73, -2.06)	7.6716 (6.86, 8.48)	2.2003 (1.71, 2.69)
NM_001979	*EPHX2*	Epoxide hydrolase 2, cytoplasmic	-5.9138 (-6.12, -5.71)	2.5701 (2.49, 2.65)	4.2775 (3.83, 4.73)	2.7388 (2.08, 3.39)	1.6439 (1.21, 2.07)
NM_000502	*EPX*	Eosinophil peroxidase	-8.7423 (-8.86, -8.63)	-1.1168 (-1.42, -0.81)	-1.4692 (-2.21, -0.73)	1.0843 (0.83, 1.34)	-1.7724 (-1.90,-1.65)

NM_021953	*FOXM1*	Forkhead box M1	-6.8878 (-7.67, -6.10)	-1.6907 (-1.91, -1.47)	1.4857 (0.73, 2.24)	-1.661 (-2.18, -1.14)	-1.1241 (-1.38, -0.87)
NM_002032	*FTH1*	Ferritin, heavy polypeptide 1	-5.5031 (-6.58, -4.43)	1.8668 (1.70, 2.04)	1.1596 (0.88, 1.44)	3.8762 (3.58, 4.18)	1.5614 (0.75, 2.38)
NM_001498	*GCLC*	Glutamate-cysteine ligase, catalytic subunit	-6.7598 (-7.89, -5.63)	-1.2325 (-1.48, -0.99)	-1.815 (-1.98, -1.65)	-1.1568 (-2.14, -0.17)	-1.3653 (-1.59, -1.14)
NM_002061	*GCLM*	Glutamate-cysteine ligase, modifier subunit	-14.8016 (-15.7, -13.9)	1.7915 (1.35, 2.23)	1.0787 (0.55, 1.61)	2.7781 (1.99, 3.56)	1.0141 (0.32, 1.71)
NM_000581	*GPX1*	Glutathione peroxidase 1	-9.9476 (-11.1, -8.75)	-1.3381 (-1.65, -1.02)	-1.3237 (-1.96, -0.69)	1.4108 (1.13, 1.70)	-1.8694 (-2.06, -1.68)
NM_002083	*GPX2*	Glutathione peroxidase 2 (gastrointestinal)	-4.2246 (-4.48, -3.96)	1.2071 (1.05, 1.36)	-1.5539 (-1.69, -1.41)	2.6621 (2.65, 2.68)	-1.547 (-1.94, -1.15)
NM_002084	*GPX3*	Glutathione peroxidase 3 (plasma)	-1.1494 (-1.24, -1.06)	2.1856 (1.85, 2.52)	1.0602 (0.97, 1.15)	-2.5149 (-3.49, -1.54)	1.6969 (1.55, 1.85)
NM_002085	*GPX4*	Glutathione peroxidase 4 (phospholipid hydroperoxidase)	-2.5022 (-2.60, -2.41)	-1.6807 (-2.04, -1.33)	2.8301 (2.30, 3.36)	1.2518 (0.84, 1.67)	1.0475 (0.55, 1.55)
NM_001509	*GPX5*	Glutathione peroxidase 5 (epididymal androgen-related protein)	-1.218 (-2.55, 0.12)	1.2443 (1.24, 1.245)	2.0054 (1.09, 2.93)	2.5355 (2.34, 2.74)	1.4536 (0.87, 2.03)
NM_182701	*GPX6*	Glutathione peroxidase 6 (olfactory)	-1.218 (-2.55, 0.12)	1.2443 (1.24, 1.245)	2.0054 (1.09, 2.93)	2.5355 (2.34, 2.74)	1.4536 (0.87, 2.03)
NM_015696	*GPX7*	Glutathione peroxidase 7	-4.2152 (-4.85, -3.58)	1.2294 (0.95, 1.51)	3.8358 (3.74, 3.93)	1.7798 (0.95, 2.61)	1.684 (0.68, 2.68)
NM_000637	*GSR*	Glutathione reductase	-8.8333 (-9.53, -8.14)	-1.7181 (-2.60, -0.84)	1.0164 (0.20, 1.83)	1.0321 (0.88, 1.18)	1.2323 (0.98, 1.48)
NM_000178	*GSS*	Glutathione synthetase	-3.888 (-4.37, -3.40)	1.6643 (1.27, 2.06)	1.6297 (1.56, 1.69)	1.9683 (1.76, 2.18)	1.0711 (0.95, 1.19)
NM_000852	*GSTP1*	Glutathione S-transferase pi 1	-4.5394 (-5.24, -3.84)	1.0531 (0.60, 1.50)	1.3586 (0.46, 2.26)	1.3872 (0.73, 2.04)	1.2971 (0.47, 2.13)
NM_001513	*GSTZ1*	Glutathione transferase zeta 1	-5.8241 (-6.73, -4.92)	1.2814 (0.53, 2.03)	1.589 (1.24, 1.93)	2.0829 (1.69, 2.48)	-1.1855 (-1.36, -1.01)
NM_001518	*GTF2I*	General transcription factor IIi	-3.2004 (-3.37, -3.03)	1.0899 (0.80, 1.38)	-1.1165 (-1.30, -0.94)	1.6082 (1.33, 1.89)	1.2143 (0.84, 1.59)
NM_002133	*HMOX1*	Heme oxygenase (decycling) 1	-17.4205 (-18.9, -15.9)	2.9286 (2.77, 3.08)	1.0773 (0.91, 1.25)	-4.7073 (-6.67, -2.74)	1.5214 (1.11, 1.93)
NM_005345	*HSPA1A*	Heat shock 70kDa protein 1A	-2.5344 (-2.61, -2.46)	3.5447 (3.23, 3.86)	1.8268 (1.62, 2.04)	1.1495 (0.78,1.52)	2.7246 (1.95, 3.50)
NM_006121	*KRT1*	Keratin 1	-1.218 (-1.48, -0.95)	1.2443 (1.24, 1.245)	2.0054 (1.09, 2.93)	2.5355 (2.34, 2.74)	1.4536 (0.87, 2.03)
NM_006151	*LPO*	Lactoperoxidase	1.9792 (1.68, 2.28)	1.0895 (0.88, 1.30)	1.9596 (1.46, 2.46)	2.2202 (2.02,2.42)	1.4101 (0.92, 1.91)
NM_005368	*MB*	Myoglobin	-1.3113	4.7115	3.0604	11.4259	1.3744

			(-2.0, -0.62)	(3.84, 5.58)	(2.87, 3.25)	(10.2,12.6)	(1.35, 1.40)
NM_000242	*MBL2*	Mannose-binding lectin (protein C) 2, soluble	-1.218 (-1.48, -0.95)	1.2443 (0.68, 1.81)	2.0054 (1.09, 2.93)	2.5355 (2.34, 2.74)	1.4536 (0.87, 2.03)
NM_004528	*MGST3*	Microsomal glutathione S-transferase 3	-2.5642 (-2.9,-2.23)	1.0542 (0.89, 1.22)	2.9898 (2.91, 3.07)	1.7291 (1.20, 2.26)	1.6425 (1.56, 1.73)
NM_000250	*MPO*	Myeloperoxidase	-1.2974 (-1.38, -1.22)	3.3802 (2.96, 3.80)	-1.0158 (-1.25, -0.78)	4.0338 (3.73, 4.34)	1.1291 (0.79, 1.47)
NM_002437	*MPV17*	MpV17 mitochondrial inner membrane protein	-1.6822 (-1.85, -1.51)	-1.3307 (-1.75, -0.91)	1.3364 (1.01, 1.66)	1.6156 (0.91, 2.32)	1.0739 (0.81, 1.34)
NM_012331	*MSRA*	Methionine sulfoxide reductase A	-1.8697 (-2.08, -1.66)	-1.157 (-1.33, -0.98)	1.3508 (0.77, 1.93)	1.014 (0.85,1.18)	1.3726 (0.87, 1.87)
NM_005954	*MT3*	Metallothionein 3	1.273 (0.57,1.98)	4.3129 (4.17, 4.46)	1.415 (0.40, 2.43)	3.4754 (2.35, 4.60)	1.1101 (0.75, 1.47)
NM_000265	*NCF1*	Neutrophil cytosolic factor 1	1.0959 (0.60, 1.60)	2.3434 (1.74, 2.95)	1.3578 (1.23, 1.48)	3.3824 (2.85, 3.91)	1.9107 (1.75, 2.07)
NM_000433	*NCF2*	Neutrophil cytosolic factor 2	-2.0103 (-2.30, -1.73)	-1.204 (-1.45, -0.96)	-1.0394 (-1.38, -0.70)	1.6159 (1.38,1.85)	1.0585 (0.39, 1.73)
NM_000625	*NOS2*	Nitric oxide synthase 2, inducible	-1.4552 (-2.31, -0.60)	-1.8079 (-2.10, -1.52)	-2.5416 (-2.56, -2.52)	-3.9609 (-4.33, -3.59)	-2.3773 (-2.95, -180)
NM_016931	*NOX4*	NADPH oxidase 4	-1.1886 (-1.39, -0.99)	1.2443 (0.68, 1.81)	2.0054 (1.93, 2.08)	2.5355 (2.34, 2.74)	1.4536 (0.87, 2.03)
NM_024505	*NOX5*	NADPH oxidase, EF-hand calcium binding domain 5	-1.1189 (-2.08, -0.16)	1.2129 (0.72, 1.70)	1.9549 (1.88, 2.03)	2.8305 (2.63, 3.03)	1.417 (0.87, 2.00)
NM_000903	*NQO1*	NAD(P)H dehydrogenase, quinone 1	-1.0643 (-1.88, -0.25)	-2.0577 (-2.30, -1.82)	-1.193 (-1.78, -0.61)	1.566 (0.90,2.23)	1.2073 (0.52, 1.90)
NM_002452	*NUDT1*	Nudix (nucleoside diphosphate linked moiety X)-type motif 1	-1.4977 (-1.64, -1.35)	-4.3326 (-4.42, -4.24)	-2.538 (-3.18, -1.90)	-1.2537 (-1.63, -0.84)	1.2001 (0.11, 2.29)
NM_181354	*OXR1*	Oxidation resistance 1	-1.8265 (-2.08, -1.58)	-1.0454 (-1.23, -0.86)	1.7374 (1.38, 2.09)	2.1706 (1.87,2.47)	1.2382 (0.62, 1.85)
NM_005109	*OXSR1*	Oxidative-stress responsive 1	-1.7917 (-2.97, -0.61)	-2.7743 (-2.97, -2.58)	1.0338 (0.54, 1.53)	1.0727 (0.64,1.51)	1.3344 (0.94,1.73)
NM_020992	*PDLIM1*	PDZ and LIM domain 1	-1.1614 (-1.43, -0.89)	1.6735 (1.57, 1,78)	1.12 (0.73, 1.51)	1.2009 (0.72,1.68)	1.1893 (0.98, 1.40)
NM_007254	*PNKP*	Polynucleotide kinase 3'-phosphatase	-1.1021 (-1.25, -0.96)	1.1907 (1.00, 1.38)	-1.0024 (-1.18, -0.83)	1.5302 (1.36, 1.70)	1.1741 (1.05, 1.30)
NM_002574	*PRDX1*	Peroxiredoxin 1	-1.0586 (-1.88, -0.24)	-1.6287 (-1.74, -1.52)	1.1151 (0.60, 1.63)	-1.3472 (-2.50,-020)	1.1002 (0.72, 1.48)
NM_005809	*PRDX2*	Peroxiredoxin 2	-1.2289 (-2.06, -0.39)	-1.7831 (- 2.36, -1.21)	-1.5443 (-1.64, -1.45)	-1.2228 (-1.32,-1.12)	-1.0604 (- 1.16, -0.96)
NM_006793	*PRDX3*	Peroxiredoxin 3	1.9735 (1.67, 2.28)	-59.9174 (-61.9, -57.9)	2.7939 (1.86, 3.73)	-31.4587 (-32.9,-29.9)	2.9527 (2.85, 3.05)

NM_006406	*PRDX4*	Peroxiredoxin 4	1.4569 (1.34, 1.58)	-9.5973 (-11.2, -7.96)	1.5685 (0.84, 2.30)	-128.2006 (-129.2, -127.2)	1.4459 (0.77, 2.12)
NM_181652	*PRDX5*	Peroxiredoxin 5	-1.7374 (-2.07, -1.41)	-16.428 (-17.2, -15.7)	-1.3749 (-1.89, -0.86)	-23.3438 (-24.7, -21.9)	-1.058 (-2.02, -0.09)
NM_004905	*PRDX6*	Peroxiredoxin 6	1.4313 (0.94, 1.92)	-7.3284 (-8.36, -6.30)	-1.0592 (-1.21, -0.91)	1.4005 (1.27,1.53)	1.0677 (0.87, 1.26)
NM_020820	*PREX1*	Phosphatidylinositol-3,4,5-trisphosphate-dependent Rac exchange factor 1	1.3102 (0.64, 1.98)	-4.2637 (-7.13, -3.40)	-1.0834 (-2.16, -0.01)	1.0593 (0.66, 1.46)	1.1031 (2.53, 1.68)
NM_183079	*PRNP*	Prion protein	-1.0184 (-1.08, -0.96)	-7.8286 (-8.85, -6.81)	-2.3873 (-2.49, -2.29)	-1.4693 (-1.75,-1.19)	-1.1675 (-1.37, -0.97)
NM_000962	*PTGS1*	Prostaglandin-endoperoxide synthase 1 (prostaglandin G/H synthase and cyclooxygenase)	-1.0174 (-1.37, -0.67)	-3.7241 (-4.82, -2.62)	2.3115 (2.25, 2.37)	1.4318 (1.35,1.52)	1.4713 (1.31, 1.63)
NM_000963	*PTGS2*	Prostaglandin-endoperoxide synthase 2 (prostaglandin G/H synthase and cyclooxygenase)	-1.6068 (-1.67, -1.54)	-2.2803 (-3.28, -1.28)	2.4398 (1.78, 3.10)	1.2189 (1.20, 1.23)	1.4338 (0.93, 1.93)
NM_012293	*PXDN*	Peroxidasin homolog (Drosophila)	-2.5606 (-2.66, -2.46)	10.9273 (10.2, 11.7)	2.7915 (2.57, 3.01)	4.3033 (3.33, 5.28)	-1.4162 (-1.48, -1.35)
NM_014245	*RNF7*	Ring finger protein 7	1.6815 (0.80, 2.56)	-1.9938 (-2.83, -1.15)	1.3337 (1.25, 1.41)	1.6521 (1.25, 2.06)	1.813 (1.66, 1.97)
NM_182826	*SCARA3*	Scavenger receptor class A, member 3	-1.0657 (-1.39, -0.75)	3.387 (3.10, 3.67)	-1.7869 (-1.99, -1.58)	2.6369 (2.08, 3.20)	1.219 (0.80, 1.64)
NM_203472	*SELS*	Selenoprotein S	1.4782 (1.40, 1.56)	-2.6199 (-2.64, -2,60)	-1.216 (-1.29, -1.14)	-1.2338 (-2.08, -0.39)	1.378 (1.23, 1.52)
NM_005410	*SEPP1*	Selenoprotein P, plasma, 1	-1.0385 (-1.12, -0.96)	1.0313 (0.70, 1.36)	-2.6258 (-2.82, -2.43)	-3.0103 (-3.66, -2.36)	-1.6685 (-2.33, -1.01)
NM_003019	*SFTPD*	Surfactant protein D	-1.1568 (-1.17, -1.14)	-4.1 (-4.17, -4.03)	-1.4399 (-1.71,- 1.17)	-5.0053 (-5.13, -4.88)	1.2388 (0.63, 1.84)
NM_012237	*SIRT2*	Sirtuin 2	-1.5626 (-2.88, -0.25)	-26.8411 (-28.1, -26.6)	-2.2367 (-2.30,-2.18)	-9.6471 (-10.69, -8.60)	-1.0538 (-1.22, -0.89)
NM_000454	*SOD1*	Superoxide dismutase 1, soluble	1.4664 (1.39, 1.55)	-10.8719 (-11.8, -9.90)	-1.5836 (-1.94, -1.22)	1.2741 (0.65, 1.90)	1.1003 (0,59, 1.61)
NM_000636	*SOD2*	Superoxide dismutase 2, mitochondrial	1.2945 (1.08, 1.51)	-3.1946 (- 4.14, -2.25)	-1.4763 (-1.53, -1.42)	2.1632 (1.43, 2.89)	1.6019 (0.90, 2.31)
NM_003102	*SOD3*	Superoxide dismutase 3, extracellular	-1.5305 (-1.61, -1.45)	2.8656 (1.72, 4.01)	1.3171 (1.24, 1.39)	2.4902 (2.32, 2.66)	-1.0488 (-1.63, -0.47)
NM_003900	*SQSTM1*	Sequestosome 1	1.4409 (0.98, 1.90)	-3.4724 (-4.74, -2.20)	2.6403 (2.48, 2.80)	2.4913 (2.01, 2.97)	1.9603 (1.83, 2.09)
NM_080725	*SRXN1*	Sulfiredoxin 1	-1.0578 (-1.38, -0,74)	-7.2786 (-8.73, -5.83)	1.6029 (1.55,165)	1.076 (0.23, 1,92)	1.173 (0.89, 1.46)
NM_006374	*STK25*	Serine/threonine kinase 25	-1.3484	-3.2342	1.314	1.037	1.3018

			(-1.46, -1.24)	(-4.32, -2.15)	(1.30, 1.33)	(0.85, 1.22)	(1.20, 1.40)
NM_000547	*TPO*	Thyroid peroxidase	-1.2831 (-1.55, -1.02)	1.7172 (0.96, 2.48)	1.9037 (1.83, 1.98)	3.3136 (3.29, 3.33)	1.3798 (0.80, 1.96)
NM_003319	*TTN*	Titin	-2.6732 (-2.89, -2.45)	2.8289 (1.90, 3.76)	1.6405 (0.74, 2.54)	7.0756 (6.25, 7.90)	-1.3269 (-1.55, -1.11)
NM_003329	*TXN*	Thioredoxin	1.3935 (0.89, 1.89)	-4.2025 (-5.15, -3.26)	1.2438 (0.40, 2.09)	1.2924 (0.60, 1.97)	1.5323 (0.99, 2.08)
NM_003330	*TXNRD1*	Thioredoxin reductase 1	1.1094 (0.88, 1.33)	-28.0526 (-29.6, -26.5)	1.0045 (0.93, 1.08)	-1.3382 (-1.37, -1.30)	1.2373 (0.66, 1.81)
NM_006440	*TXNRD2*	Thioredoxin reductase 2	-3.3648 (-4.13, -2.60)	-15.9822 (-17.1, -14.9)	-1.6794 (-2.47, -0.88)	-13.0387 (-14.3, -11.7)	1.0944 (0.70, 1.49)
NM_003355	*UCP2*	Uncoupling protein 2 (mitochondrial, proton carrier)	-2.783 (-2.93, -2.64)	-106.3798 (-107.5, -105.2)	-4.5572 (-4.99,-4.12)	-5.7665 (-5.93,-5.60)	-2.2297 (-2.39, -2.07)

[a] Regulation of gene expression was measured after 2 days of incubation with 13 µM BZ using the RT^2 Human Oxidative Stress PCR expression array analysis (QIAGEN). Data are represented as fold change Data are represented as fold change and 95 % confidence interval (95% CI).
